# Changes in population susceptibility to heat and cold over time: assessing adaptation to climate change

**DOI:** 10.1186/s12940-016-0102-7

**Published:** 2016-03-08

**Authors:** Katherine Arbuthnott, Shakoor Hajat, Clare Heaviside, Sotiris Vardoulakis

**Affiliations:** Department of Social and Environmental Health Research, London School of Hygiene & Tropical Medicine, London,, WC1H 9SH UK; Environmental Change Department, Centre for Radiation, Chemical and Environmental Hazards, Public Health England, Didcot, OX11 0RQ UK

**Keywords:** Climate change, Adaptation, Temperature, Heat, Cold, Heatwave, Mortality, Health

## Abstract

**Background:**

In the context of a warming climate and increasing urbanisation (with the associated urban heat island effect), interest in understanding temperature related health effects is growing. Previous reviews have examined how the temperature-mortality relationship varies by geographical location. There have been no reviews examining the empirical evidence for changes in population susceptibility to the effects of heat and/or cold over time. The objective of this paper is to review studies which have specifically examined variations in temperature related mortality risks over the 20^th^ and 21^st^ centuries and determine whether population adaptation to heat and/or cold has occurred.

**Methods:**

We searched five electronic databases combining search terms for three main concepts: temperature, health outcomes and changes in vulnerability or adaptation. Studies included were those which quantified the risk of heat related mortality with changing ambient temperature in a specific location over time, or those which compared mortality outcomes between two different extreme temperature events (heatwaves) in one location.

**Results:**

The electronic searches returned 9183 titles and abstracts, of which eleven studies examining the effects of ambient temperature over time were included and six studies comparing the effect of different heatwaves at discrete time points were included. Of the eleven papers that quantified the risk of, or absolute heat related mortality over time, ten found a decrease in susceptibility over time of which five found the decrease to be significant. The magnitude of the decrease varied by location. Only two studies attempted to quantitatively attribute changes in susceptibility to specific adaptive measures and found no significant association between the risk of heat related mortality and air conditioning prevalence within or between cities over time. Four of the six papers examining effects of heatwaves found a decrease in expected mortality in later years. Five studies examined the risk of cold. In contrast to the changes in heat related mortality observed, only one found a significant decrease in cold related mortality in later time periods.

**Conclusions:**

There is evidence that across a number of different settings, population susceptibility to heat and heatwaves has been decreasing. These changes in heat related susceptibility have important implications for health impact assessments of future heat related risk. A similar decrease in cold related mortality was not shown. Adaptation to heat has implications for future planning, particularly in urban areas, with anticipated increases in temperature due to climate change.

**Electronic supplementary material:**

The online version of this article (doi:10.1186/s12940-016-0102-7) contains supplementary material, which is available to authorized users.

## Background

The global climate is projected to warm although to what extent depends on future greenhouse gas emissions and socioeconomic and land use changes. Global surface temperatures are likely to warm by between 0.3 °C and 4.8 °C by the end of this century relative to the end of the last, depending on modelling choices which reflect differences in the amount of anthropogenic forcing in different scenarios [1]. It is anticipated that there will be increasing variability in future temperatures and extreme weather events over most geographical regions [1–4]. For example, heatwaves are likely to increase in frequency and severity and this, combined with projected demographic changes, will lead to an increase in population exposure to extreme events [5, 6]. However, the same locations may still experience (extreme) low temperatures. These are important considerations for public health, as both heat and cold exposure lead to increased risk of mortality [7–21].

**Fig. 1:**
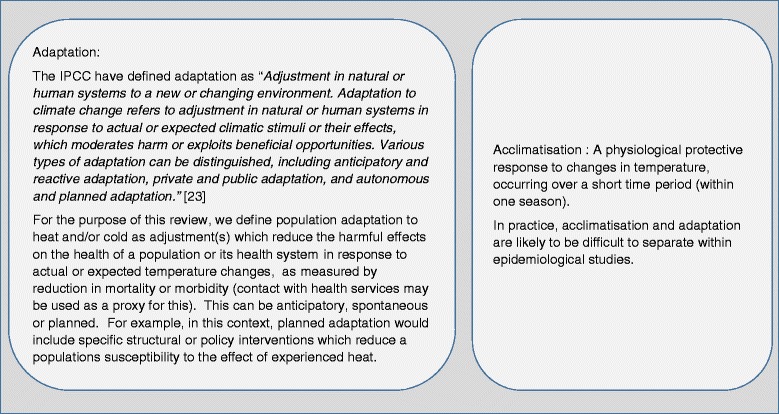
Definition of Adaptation (based on the Intergovernmental Panel on Climate Change (IPCC) definition [23]) and Acclimatisation

Adequate public health responses to temperature related effects of climate change require a sound risk management process, informed by the use and synthesis of relevant evidence. A framework for such a public health approach for climate change adaptation is outlined by Hess et al. [22]. In considering the future impact of temperature on health, knowledge about past and current risks to health from changes in ambient temperature is essential: it informs the baselines used for future risk assessments upon which management strategies may be based. Changes in temperature related health outcomes over time could give valuable insight into whether populations have adapted to hot and/or cold temperatures in more recent times. Understanding what has caused changes in susceptibility to temperature related mortality can help inform current public health policy and protection of vulnerable communities. Alternatively, if temperature related mortality remains unchanged this gives further weight to the need for specific planned adaptive strategies to address the health risks of future climate change. For the purpose of this review, adaptation and acclimatisation have been defined as in Fig. 1 below, with the definition of adaptation based upon that of the Intergovernmental Panel on Climate Change [23]. However, a distinction between evidence of decreasing susceptibility to heat and cold and evidence that adaptation or acclimatisation have occurred should be made. For example, a decrease in temperature related mortality may have arisen through general improvements in health or social care rather than specific planned adaptations to the effects of heat or cold: to attribute decreasing heat or cold related mortality solely to planned adaptive measures would be misleading.

Epidemiological evidence for the effect of temperature on health outcomes is typically based on observational studies. The relative risk of mortality per unit change in temperature (e.g. per degrees Celsius (°C)) is generally estimated using a time series or case-crossover approach. This is usually denoted by ‘U’, ‘V’ or ‘J’ type curves, with adverse health effects appearing below or above a given range of temperatures [11]. Where a threshold temperature is set, above or below which health effects occur (and can be estimated using a log-linear or non-linear approach), this point is often referred to as the Minimum Mortality Temperature (MMT). The effect of individual heatwaves is often estimated using episode analysis, where observed numbers of deaths during the heatwave period are compared to expected deaths estimated using an appropriate baseline.

A number of epidemiological studies [24–26] have examined how temperature-mortality relationships vary by geographical location. The geographical variation in this relationship is also the subject of a review by Hajat and Kosatsky [27], who explored possible explanations for the differences in temperature related susceptibility between countries. In a random-effects meta-regression of studies, the relative risk of heat related mortality was found to be strongly related to heat thresholds. Heat thresholds (and RR of heat-related mortality) were higher in countries closer to the equator (with higher summertime mean temperatures). It was proposed that the higher thresholds seen in countries closer to the equator, may indicate some level of population adaptation to heat. The risk of heat-related mortality was also found to increase with increasing urban density, decreasing city level GDP and increasing age of the population.

No review, however, has examined how or whether temperature-related mortality varies over time in one location. This paper seeks to address this gap in knowledge. Specifically we review the evidence for changing population susceptibility (in terms of mortality) to ambient heat and cold and heatwaves or cold snaps over different time points over the last century and more recently. Understanding changing temperature-related mortality, the time scales over which this has occurred, and its possible causes could make important contributions to managing future risk. We discuss the extent to which changes in susceptibility are attributed to planned adaptive measures within the selected studies and consider how this evidence could be used in assessments of future temperature related health impacts. Both heat and cold related mortality are reviewed, as in many parts of the world studies suggest cold related mortality currently has and will continue to have a substantial contribution to temperature related mortality, even under warming projections [28, 29].

We review both changes in mortality in response to general temperature increases or decreases and to extreme weather events, such as heatwaves and cold snaps. Extreme events are included since the specific adaptive measures and policies relating to these may differ to those for general temperature effects. For example, there are many specific measures, such as heat health warning systems (HHWS) that are only fully activated during an extreme event [30, 31]. Political will to react to extreme events, such as the 2003 heatwave (commonly stated as the trigger for many European countries’ HHWS) may be greater [32], as although considered low probability they have an immediate and high impact compared to slowly changing environmental risk.

Only the direct effects of ambient temperature on health (all cause and cause specific mortality – for example mortality due to cardiac or respiratory disease) are considered in this review. A review of individual and specific adaptive measures (e.g. the effectiveness of electric fans, or heat health warning systems) is beyond the scope of this paper and has, in part, been undertaken in previous works [33–35].

## Methods

All populations, analysed/aggregated at either city, regional or national level, were included in this review. We included observational studies (time series, case-crossover or period analysis design) which:

- quantified the risk of health related events with changing ambient temperature in one location over a given time period (not limited); or
- compared outcomes between two different discrete extreme temperature events (>1 day, for example, usually defined by the context specific definition of a heatwave or cold spell) in one location.

Where studies compared the effect of temperature extremes but by individual days (e.g. risk at the 98^th^ percentile of temperatures compared with average temperature but as part of a heatwave) these were categorised as the first type of study – assessing the effect of ambient increased temperature on health.

The primary outcome assessed was mortality (all cause or by type), as estimations of this are not sensitive to changes in organisation of care (whereas, hospital admission rates for example, may change over time, not as a function of morbidity but related to changing expectations or access to care). Studies which only examined deaths coded as due to heat or temperature disturbances (e.g. heatstroke, hypo/hyper-thermia) were excluded as these deaths are comparatively rare, the coding of such death may vary and they may also be associated with occupational or working conditions unrelated to ambient temperature (e.g. heat stroke may occur in military recruits in training etc.). Studies were excluded if there were no quantitative results available that compared mortality (risk or rates or attributable burden) over time.

Five electronic databases were searched (Ovid MEDLINE, Ovid EMBASE, CINAHL, Psych- info and Global Health) using three main concepts: temperature, health outcomes and changes in vulnerability or adaptations. Search terms were combined using the appropriate Boolean operator terms and limited to English and to humans. Further articles were identified through snow-balling of references and hand searching of relevant journals not indexed in the databases (e.g. Nature Climate Change).

Data from studies was extracted on location and duration of the study, exposures studied, health outcome measures, methods used for estimating the effect and methods used to assess changes in mortality at the time points recorded. Where available, subgroup analysis was also recorded (e.g. by age category or by cause of death). Contextual information, for example whether protective measures had been introduced during the study time period, was recorded even if the description of these was qualitative rather than quantitative.

Due to the heterogeneity of approaches to defining and assessing changes in temperature related mortality risk (for example, changes in relative risk (RR) or attributable mortality burdens over time) a meta-analysis was not deemed appropriate. Where complete results from more than one statistical model were presented, those that were reported in full or stated to be the main model by the authors are included. When results from more than one model were given, those judged to have the best control for confounders or best fit to data were chosen. Where estimates were made over a period of time the mid-point of this time period was used when representing the information.

## Results

Eleven studies met the inclusion criteria examining changes in susceptibility to heat and cold over time and six studies of heatwaves met the inclusion criteria.

### Changes in vulnerability to ambient heat and cold over time (non- heatwaves)

#### Types of study and methods used

Eleven studies [36–46] were identified that had quantitatively analysed changes in the effects of either ambient heat, cold or both on mortality over time. The key information about study populations, outcomes and methods is summarised in Table 1. The majority of studies used data from the US or Europe. The time periods studied ranged from 18 to 150 years. Eight studies focused only on urban populations [36–40, 43, 46], eight analysed all age groups of which four reported trends in time also by age category [36–39] and two papers only analysed older age groups [43, 45]. Five studies examined the effects of both high and low temperatures [39, 41–44], whilst all others only examined the effect of heat. Ten papers examined all-cause mortality, of which three also analysed trends in heat related cardiovascular and/or respiratory deaths [37, 38, 44] and one paper only analysed cardiovascular mortality [43].

**Table 1:** Characteristics and results of studies analysing temporal changes in temperature related mortality

Study	Location time period population	Exposure(s) and outcomes	General modelling approach and methods to assess change in susceptibility over time	Results: changes in (RR) of heat/cold related mortality (HRM, CRM) over time (all CI/PIs and significance are for 5 % level unless stated otherwise)
Bobb et al. 2014 [37]	105 US cities 1987–2005 All ages & age stratified	Heat (only summer months) All-cause mortality & CVD / Respiratory mortality	Time series regression (daily series) model. Control for time varying factors. Estimated excess heat related deaths for each year (1987 and 2005 results compared). Each year allowed a separate coefficient for daily temperature.	Heat related deaths per 1000 deaths (all cities):51 (95 % PI: 42,61) in 1987 compared to 19 (95 % PI: 12,27) in 2005. Decline observed for all ages & significant for heat related respiratory & CVD mortality. Cities with larger increases in AC had larger decreases in mortality (not significant).
Petkova et al. 2014 [36]	New York (US) 1900–1948 & 1973–2006 All ages & age stratified	Heat (only summer months) All-cause mortality	Time series regression (daily series). Control for time varying factors. Modelled risk of mortality at 29 °C vs 22 °C for each decade. Decadal averages of RR at 29 °C vs 22 °C compared. Used random effects meta-regression, including linear term for decade.	Decrease in RR at 29 °C vs 22 °C of 4.6 % (2.4,6.7) per decade (all ages) >65 years: highest initial risk and most decline in RR over time. Also found a change in lag structure over time - harvesting effect more prevalent in earlier part of century.
Astrom et al. 2013 [39]	Stockholm, Sweden 1901–2009 All ages & stratified by age and sex	Heat and cold ‘extremes’ (Defined in model 1 as above/below the 98^th^ percentile for entire period) Daily mortality	Time series regression (daily series). Control for time varying factors. Examined trend in RR of mortality at extremes of temperature over time of mortality at 98th percentiles of temperature compared to mortality at average temperatures.	Significant decline in mortality risk for elderly and combined age categories for heat but non-significant for cold. Patterns similar for men & women Significant declining trend in temperature related mortality risk for 0-14 s for hot and cold. In last decades, upward trend in the heat risk for the 15–64 age group observed.
Ha et al. 2013 [38]	Seoul, S. Korea 1993–2009 (1994 excluded: extreme HW) All ages & age stratified	Heat All-cause mortality (excluding accidental deaths) and CVD mortality	Time series regression (daily series). Linear threshold model to estimate quantitative effects. Control for time varying factors. Compared results from two periods (1993 and 1995–2000, and 2001–2009). Used common threshold throughout study period.	% increase in all-cause mortality per 1 °C increase in temperature above threshold (changes not significant): All-cause mortality (pattern similar for >65s) 1990s 4.73 % (all ages) 2000s 6.05 % (all ages) CVD mortality (pattern similar for >65s) 1990s 8.69 % (all ages) and 2000s (all ages) 5.27 %
Matzarakis et al. 2011 [40]	Vienna, Austria 1970–2007 All ages	Heat (Physiological Equivalent Temperature (PET)) All-cause mortality	Time series analysis (daily series). Modelled daily excess mortalities, calculated as deviations from average annual mortality. Linear regressions fitted to mortality rates per 10000 to give % change in heat related mortality per decade (1970–2007) for given ranges of PET.	% change per decade from 1970 to 2007 in mortality: PET range <29 °C - 0.15 %: ( reported not significant) PET range 29-35 °C −0.83 % (−0.68,-0.97) PET range 35-41 °C −0.96 % (−0.77,-1.16) PET range > =41 °C −1.32 % ( not significant - low numbers)
Christidis et al. 2010 [41]	England and wales 1976–2005 All ages	Heat and cold All-cause mortality	Daily excess HRM/CRM obtained by comparing to the average mortality within a 3 °C ‘comfort zone’. Compared: 1.yearly regression slopes (1976–2005) 2.Change in HRM/CRM obtained using regression slopes from different time periods (1976 compared to 2005) to demonstrate no adaptation or early adaptation.	Slope of regression lines for heat and cold related mortality risk (SE) decreased in magnitude over time. CRM decreased by 85 deaths/million/year from 1976–2005. “No adaptation” scenario (1976 regression slope) CRM reduction less 47 deaths/million/year. HRM increased by 0.7 deaths/ million/ year. “No adaptation” scenario (1976 slope) HRM increased more (by 1.6 deaths/million/year).
Ekamper, 2009 [42]	Zeeland, Holland 1855–2006 All ages & age stratified	Heat and cold All-cause mortality	Times series analysis (daily series) Compare: a) regression co-efficient from model between 25 year periods (thresholds allowed to vary between time periods) b) MMT value in each 25 year time period analysed.	Regression coefficients for HRM reported as decreasing over time (no test for significance). Pattern unclear for cold. Found shift in MMT to higher temperatures in later time periods analysed: MMT slightly below 15 °C for 1855–1897 and around 17 °C for 1905–1929 and 1930-1954
Barnett, 2007 [43]	107 US cities 1987–2000 ‘Elderly’ (age range not given)	Increases in temperature in both summer and winter (effects of heat & cold) CVD mortality	Case-crossover design Time stratified Compare the % increase in of cardiovascular deaths per 10 °F increase in temperature within a given season and across the time period 1987–2000.	% increase in risk per 10 °F rise in temperature summer Winter 1987 4.7 % (3.0, 6.5 %) 1987–4.2 (−5.1,-3.2) 2000–0.4 % (−3.2,2.5) 2000–4.9 (−6.8,-3.1) Variation between geographic regions (e.g. biggest declines in heat risk in NW, NE, Industrial MW and California)
Carson et al. 2006 [44]	London (UK) 1900–1996 All ages	Heat and cold All-cause mortality and CVD and respiratory mortality	Time series regression (weekly series). Linear hockey stick model. Controlled for time varying factors. Threshold set at 15 °C. Compared a)decadal RR for heat and cold related mortality b)proportion of deaths attributable to heat/cold.	RR (for heat related mortality above threshold) and % attributable deaths: increased between 1910 and 1937 then decreased for last 2 time points.
Davies et al. 2003 [46]	28 major US cities 1964–1998 Age standardised population	Heat only All-cause mortality	Time series analysis (daily series) using HRM: daily mortality anomalies estimated using median mortality for given month as a baseline. Analysed daily fluctuations in excess mortality with temperature variation. Compared decadal HRM. Threshold varied by decade.	Mean decadal HRM in standard population of 1 million for all cities declined over time. 12 cities showed no evidence of threshold AT above which heat related mortality begins to appear in the 1990s. Most decline in 1980s in the South in NE cities. Seattle and Washington show increased HRM in latest decades compared to the 1960s.
Donaldson et al. 2003 [45]	North Carolina (NC), South East England (SEE) South Finland (SF) 1971–1997 Age: > 55 yrs	Heat only All-cause mortality	Time series analysis using HRM (daily mortalities at daily temperatures exceeding a 3 °C threshold band, minus daily mortalities in that 3 °C band for the given month. Summed to give annual heat related mortality) Compared a) Change in temperature at which minimum mortality occurs (MMT) b) Change in excess heat related mortality per 10^6 between 1971 and 1997.	Changes in MMT (between 1971 and 1996): Increase in MMT significant for NC and SEE but not for SF Change between 1971–1996 in HRM per 10^6 population (unadjusted for age & sex and adjusted): NC 228 in 1971 decreased to 16 in 1996. Change of 212 (59,365). Adjusted change 552 (significant) SF 382 in 1971 decreased to 99 in 1996. Change of 282 (66–500). Adjusted decrease 414 (significant) SEE 111 in 1971 decreased to 16 in 1996. Change of 2.1 (−119, 114). Adjusted decrease 53 (significant)

A variety of health outcome measures were used within the time series studies to analyse the effect of temperature on health and how this varied with time (see Tables 1 and 2). Results were either presented as the RR of mortality per 1 °C (or 10 °F) increase in temperature [36, 38, 39, 43, 44], the RR of mortality at one temperature compared to another (e.g. 29 °C vs 22 °C) [36] or the 98^th^ centile vs average temperature [39] or as the (average) annual number of excess heat or cold related deaths as a proportion of the population [45, 46] or of deaths [37]. The most common approach used to examine changes in susceptibility over time was the comparison of RR or excess temperature related deaths from the models on an annual or decadal basis or between two defined time points. The extent to which trends could be identified or were quantified varied, with some studies also analysing year or decade as a modifying factor in the relationship or using regression to examine the effect of time on heat/cold related health outcomes [36, 45].

**Table 2:** Characteristics and results of studies comparing effects of heat-waves on mortality

Study	Population: location & study time periods	Definition of heat wave (HW)	Outcome measure	Methods used to compare effect of heat waves	Standardisation of HW characteristics?	Results: health outcomes	Comments and explanations given for changes in mortality between events
Kysely et al. 2012 [51]	Czech Republic 1986-2006	≥2 days with temperature >95^th^ quartile of distribution for given part of the year	All-cause and CVD mortality	Determined whether the deviation of observed deaths significant compared to expected deaths estimated by Monte Carlo method using data drawn from summers between 1986–2006.	Within common definition, length & intensity of HW allowed to vary between years.	Linear test for trend for deviation of mortality for hot spells between 1986 and 2009. Decrease in mortality over time found (significant at p = 0.05 level). Decline of around 0.4-0.5 % deaths per year.	Hypothesised decreasing mortality due to acclimatisation to heat within a summer season in later years and/or increased adaptive measures such as improved living, health & building standards and increased heat awareness
Kysely et al. 2008 [52]	Czech Republic 2003 HW compared to period 1986-2006	≥3 days with average daily heat index exceeding 95 % quartile of distribution and ≥ 1 day exceeding 98 % quartile	All-cause and CVD mortality	Observed and expected mortality compared. Expected deaths over April-September period computed using smoothed 15 day running means corrected for weekly cycle and annual changes in mortality .	Within common definition, length & intensity of HW allowed to vary between years.	Taken together, the HW effects of 2003 were weaker than HW effects in previous years	Hypothesised that decreased effects of 2003 HW could be due to: factors unrelated to adaptation – e.g. influenza epidemic affecting European countries in spring 2003 reducing number of susceptible individuals or improved response to heat
Feuillet et al. 2008 [53]	France (all regions) 2006 compared to previous 29 years	2006 HW defined as period with consecutive days of alert in at least one (of 96) departments of France	All-cause mortality	Observed and expected mortality compared. Expected mortality derived from baseline deaths predicted by model using data from previous 29 years: model included seasonal control and long-term mortality trend.	Modelled expected deaths from 2006 HW using model & actual deaths from 2006 HW using mortality figures.	4388 fewer deaths than estimated by predictive model for the 2006 HW Larger decrease in the over 75 years	Hypothesised heat wave plans instigated post 2003 led to a decrease in heat wave related mortality.
Tan et al. 2007 [54]	Shanghai 2003 and 1998	≥3 days where daily maximum temperature exceeds 35 °C	All-cause mortality	Average number of deaths on heat days and non-heat days compared. Linear regressions run for 1998 and 2003 summers including mortality, temperature and air pollution concentrations to assess effect of length of HW, timing in summer and pollution.	Within common definition, length & intensity of HW allowed to vary between years.	Absolute deaths: 1998: Average number deaths on non-heat days 244, heat days 358 2003 Average number deaths on non-heat days 223, heat days 253 Not adjusted for population size/age	Hypothesised decreased HW effects could be due to: Urban green area increasing from 19.1 % to 35.2 % over the time period. Increased use of air conditioning and implementation of heat/health watch warning system in 2002
Rey et al. 2007 [55]	France (all regions) Six Heat Wave periods between 1971 and 2003	≥3 days where max and min temp simultaneously greater than respective 95^th^ percentile	All-cause and cause-specific mortality	Observed and expected mortality ration (O/E) compared for each HW Expected mortality calculated from observed mortality in previous 3 years using log-linear Poisson model of mortality rates (by month, year, age, gender, cause of death).	Within common definition, length and intensity of HW allowed to vary between years.	Observed-Expected (O-E) mortality (all cause) 1975 2952 1976 5116 1983 1473 1990 1624 2001 1330 2003 13734	In all six heatwaves, age >75 years were most vulnerable. Mortality standardised by age and gender
Smoyer et al. 1998 [56]	St Louis, Missouri 1980 and 1995 heat waves	Days with Apparent Temperature > 40.6 °C (cut off for US National Weather service warnings)	All-cause mortality	Mortality –heat relationship modelled using Poisson regression, including terms for HW duration, temperature and interaction between heat wave duration and timing in season. Best models for 1980 and 1995 selected Only > 65 years studied.	Simulated severe HW using 2 models: Model 1: deaths estimated using 1980 weather data and 1980 model parameters (adjusted for 1995 population size) Model 2:deaths estimate using 1980 weather data and 1995 model parameters	For a simulated HW: vulnerability increased using 1995 model parameters (estimated number of deaths using 1980 parameters 446 (419,465) compared to 1995 model parameters (estimated number of deaths 481 (319,822)	Imprecise estimates make the difference between 1995 and 1980 models difficult to assess .Between 1980 and 1995 the numbers of persons in the eldest age category and of older persons below the poverty line increased. Air conditioning prevalence: 1980 64.1 %, 1991 86.7 %

Where the time series models used a linear-threshold approach to estimate the effect of temperature on mortality, different decisions were taken regarding setting the threshold above or below which temperature effects were estimated. In some cases [42, 45] a change in threshold or MMT was used to support evidence for or against changes in susceptibility (i.e. an increase in threshold represents a decrease in susceptibility to heat). Even if not specifically analysed, a change in threshold is important as it relates to the slope of the regression line. One paper fixed the threshold [44] across the entire analysis period but noted that it increased in later years and two papers [42, 46, 47] allowed the threshold to vary between decades. These approaches are commented on further in the discussion section.

The amount of control for time varying factors within the epidemiological models varied. For example, only one paper specifically reported including air pollution control in the main model [44] and this was only for the last part of the century due to limited data availability (see Table 1). One study [37] reported control for air pollution as part of their sensitivity analysis and supplementary materials. In those studies reporting cold effects over time, control for influenza varied (see section on varation in effect by study design and metrics used).

#### Temporal changes in susceptibility to ambient heat

**Fig. 2:**
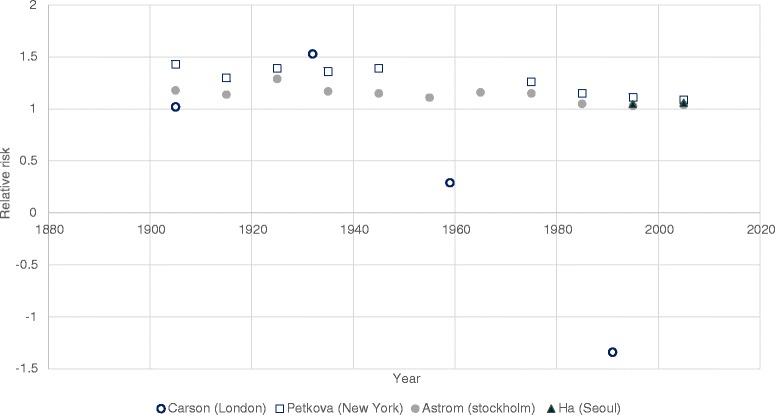
Studies reporting relative risk of heat related mortality over time. This figure shows the relative risk associated with a 1 °C increase in temperature above a common threshold (Carson et al. and Ha et al.) and the relative risk associated with extremes of high temperature compared to average temperatures (Petkova et al. and Astrom et al.). Note due to the different thresholds used, this graph is only illustrative of trends and not differences in magnitude of risk between cities

**Fig. 3:**
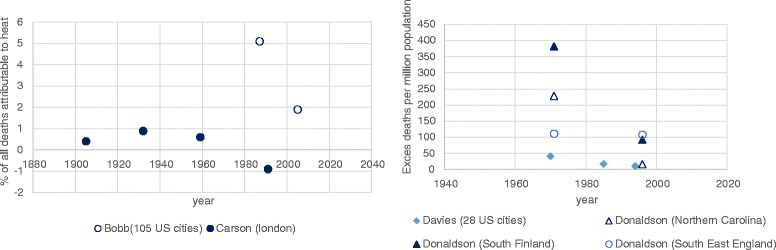
Studies reporting heat related deaths over time. This figure shows studies comparing excess heat related mortality as a proportion of all deaths (left) and studies where excess heat related mortality was reported per million population (right)

The effect of increased temperature on mortality was examined in eleven studies [36–46]. Of these, ten found evidence of some decrease in susceptibility to heat (see Table 1). Seven reported a measure of statistical significance – either a test for trend or included confidence intervals for estimates at two discrete time points. Of these seven, five found the decrease over time or between two time periods to be statistically significant at the 5 % confidence level. Given the different approaches to analysis and quantitative formulation of the outcomes, changes in RR over time are brought together graphically only for those papers which used similar methods and the same outcome metric (Figs. 2 and 3).

In those studies that examined changes in heat related mortality over the last century, most change appears to occur between the first (where risks appear substantively higher) and last part of the last century [36, 39, 44] (Fig. 2). Petkova et al. [36], appeared to show a slowing of the decrease in risk from the 1980s onwards (as the RR also approaches 1). Ha et al. [33] only analysed two points in time – both after 1990, and did not find a significant difference between RR of heat related mortality between the time points. Carson et al. [44] used larger time frames to compare risk and therefore results past 1980 cannot be visualised, however it appears that the decrease in risk after 1927 was substantial. The authors hypothesised that the large decrease seen in heat related mortality risk could be due to heat related deaths being caused by infectious diseases (such as diarrheal disease or septicaemia) in the first part of the century, but that with the epidemiological transition (the shift in burden of disease from infectious diseases to chronic non-communicable disease over time, due to improved sanitation and healthcare [48]), these have become less prominent over time. Of note, this study was the only one to use a weekly time series for the analysis of effect, which may explain some of the difference in pattern seen between this and other studies. Interestingly, Petkova et al. [36] specifically examined the effect of short term mortality displacement, and found it contributed less to heat related mortality over the last part of the century despite an ageing population.

In all studies where the proportions of deaths attributable to heat were analysed, deaths were decreased at the latest compared to earliest dates (see Table 1 and Fig. 3a and b). Two of these papers [37, 45] only presented risks for two dates, making it difficult to comment on trend. Bobb et al. [37] found the overall (combined average of all 105 US cities analysed) attributable proportion of deaths to excess heat to be significantly (5 % confidence level) less in 2005 compared to 1981. Carson et al. [44], using the same metric, also found the proportion of deaths attributable to temperatures above a given threshold to be significantly lower in the last time period compared to all others, though the pattern over the first 3 time periods is less clear. Two studies analysed deaths attributable to excess heat per million of the population (Donaldson et al. [45] and Davis et al. [46, 47]). Donaldson et al. [45] compared two specific time periods in three locations. In North Carolina and South Finland the decreases in vulnerability were significant (5 % confidence level) in all models. In South East England, the decrease was only significant in the model with control for age and sex. However, it was not possible to represent the results from the adjusted models graphically as only the changes in excess deaths were reported (i.e. no baseline or final figures) Davies et al. examined heat risk in 28 US cities [46] and showed a decreasing trend across the three time points but included no information on significance.

Four papers analysed results using different methods/outcomes to any other study and therefore are not represented graphically: Christidis et al. [41], Matzarakis et al. [40], Barnett [43] and Ekamper et al. [42].

Christidis et al. [41] investigated the hypothesis of ‘adaptation’ by comparing heat and cold related mortality estimates obtained by using regression slopes from either earlier or later years in the study. Regression slopes from earlier time periods in the study (1976) were used with weather data for the whole period to calculate heat and cold related mortality to demonstrate mortality with ‘no adaptation’. Results obtained using the slope of the regression line from later years (2005) with the same weather data as a comparison were used to demonstrate deaths accounting for ‘early adaptation’. These scenarios were compared to the actual heat and cold related mortality calculated with slopes and weather data from over the entire time period. They found actual heat related mortality increased by 0.7 deaths per million per year (using data from the whole time period) but if no adaptation had occurred heat related mortality would have increased by a larger amount (1.6 deaths per million per year over the period 1976–2005, calculated using regression slopes from the earlier time period with weather data from the whole period).

Matzaraki et al. [40], examined the change in excess mortalities attributable to different temperatures in 1970 and in 2007. For two of these ranges of temperature (29 °C to 35 °C and 35 °C to 41 °C) the excess mortality significantly decreased between the two time points. The last temperature range (>41 °C) was reported as non-significant but had low numbers of deaths.

Barnett [43] used a case-crossover approach to examine the increase in risk of cardiovascular mortality with temperature in the US. Combined estimates for all the cities showed a significant decrease in vulnerability between the two time periods analysed (1987 and 2000).

Ekamper et al. [42] reported both shifts in the MMT (which increased over time) and slopes of regression analysis. They reported a decrease in vulnerability over time but did not test significance.

#### Temporal changes in susceptibility to ambient cold

Only five studies [39, 41–44] analysed the risk of cold related deaths over time, all as part of an overall analysis of temperature related mortality (i.e. none examined cold effects alone). Results of the two of these studies which reported the RR of cold related mortality below a given threshold over time are illustrated in Fig. 2 below.

**Fig. 4:**
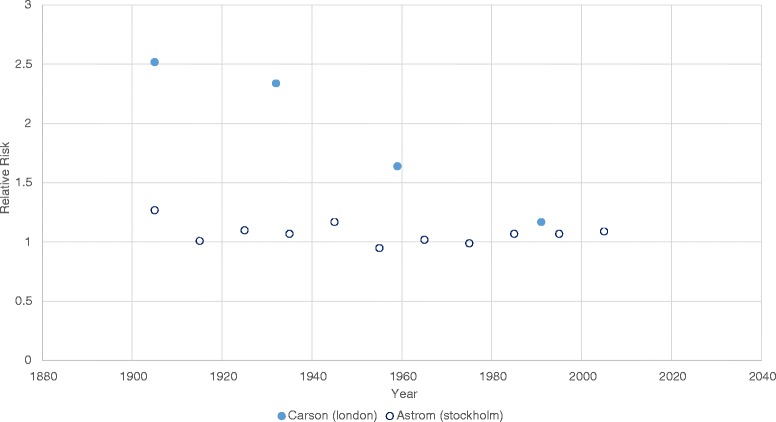
Studies reporting the relative risk of cold related mortality over time. This figure illustrates the relative risks associated with a 1 °C decrease in temperature below a common threshold

Three of the five studies examining cold effects reported decreased susceptibility over time [39, 41, 44]. Carson et al. found that this decrease was significant (at the 5 % level) in a London based study [44] (see Fig. 4 below). In a second UK based study, Christidis et al. [41], found that actual cold related mortality decreased by 85 deaths per million population per year over the period 1976–2006 (significance not reported). Using the same methods as described in the above section (on heat) to examine changes in cold related mortality under actual, ‘adaptation’ and ‘no adaptation’ scenarios they found that the decrease would have been smaller (47 deaths per million population per year) with ‘no adaptation’ (see also Table 1). Although Astrom et al. found a decrease in cold related mortality over time, it was found to be non-significant [39] except in the 0–14 year age category.

The study based in the US no clear evidence of any trend in cold related mortality over time [43] and a trend in cold related vulnerability was not clear in the study by Ekamper et al. [42].

Of note, all five studies had found a decreasing trend in heat related mortality.

One study exclusively examined the effects of cold temperatures on mortality in Spain by examining shifts in threshold for effects, but did not report quantitative results and so has not been specifically discussed in this review [49].

### Variation of results of heat and cold mortality by study characteristics

#### Variation of effect by study design and metrics used

It does not appear that the overall direction of effect over time was influenced by study design (time series, case crossover) or by the amount of time varying factors (e.g. seasonality, temporal trends, holidays etc.) controlled for by studies (see Additional file 1: Table S1a). Studies also used different approaches in either fixing the thesholds above which effects were modelled, or allowing these to vary across each time period analysed. This did not appear to alter the direction of effect demonstrated by studies (which consistently demonstrated decreasing susceptibility to heat effects regardless of precise design). However, the implications of these different choices are considered in the discussion section and in Table 3. Where sensitivity analyses were carried out, allowing definitions of extreme temperatures to vary by time periods analysed, small differences in results were seen within studies [39] (see Additional file 1: Table S1b) although the overall direction of effect remained unchanged.

**Table 3:** Advantages and disadvantages of approaches used to assess changes in susceptibility to temperature effects over time

Approach to assess change in vulnerability	Comments: advantages, disadvantages and implications of method to consider when interpreting results	Example of study using this approach
Compare minimum mortality temperature or thresholds above or below which heat/cold effects occur over time.	Simple metric for comparison. Can be determined from models using maximum likelihood estimation. Does not give information on how the RR is changing over time - an important factor in determining deaths attributable to heat or cold. The slope of the regression line is often related to the MMT or threshold. For heat effects, steeper slopes are often seen with higher thresholds. Quoting only the MMT/threshold would not include this relevant information. MMTs/thresholds can be difficult to establish with data, especially for cold – models may not always select the most appropriate threshold. It would be helpful to quote changes in MMT along with changes in RR. For example, when modelling heat effects, if there is both an increase in MMT over time and a decrease in RR despite the higher heat threshold, then this would give convincing evidence of a shift in susceptibility over time.	No included study used this approach in isolation, Carson et al. [44]. and Donaldson et al. [45] both give report MMT changes over time in addition to the changes in RR.
Compare the RR for heat or cold effects over time allowing: a) Fixing the thresholds above/below which effects are modelled over time b) Allowing thresholds above/below which effects are modelled to vary with time (e.g. allow for a shit in MMT)	Approach a: If the threshold is fixed then the changes in RR are simple and easy to interpret – i.e. allows comparison of 1 parameter only. Fixing the threshold whilst fitting a linear relationship, however, may influence results: If the threshold is fixed at a lower temperature than that at which it actually occurs, the modelled heat slope may be biased towards being more shallow. If thresholds are fixed, then giving information on how they have varied over time could help capture some information (as Carson et al. did). Approach b: Allows a better fit with the data series. However, this may lead to results which are difficult to interpret: If both measures vary then interpreting the two measures is difficult, as they are inherently related. Further, if thresholds vary by time period and only the RR is reported, this will not capture the full change in susceptibility occurring. For example, a situation may arise where both the threshold and slope have increased in a later period, but it is unclear whether the slope is artificially raised due to the higher threshold placement. Conversely, if the shape of the temperature-mortality relationship remained the same but the threshold /MMT shifted to the right over time, reporting only the RR would under-estimate the change in vulnerability. This could be misinterpreted as there being no change in susceptibility over time.	Approach a) used by Carson et al. [44] Approach b) used by Ekamper et al. [42]
Compare the RR for heat or cold effects at two defined temperatures (e.g. a) At 29 DC vs 22 DC or b) At a given percentiles of the temperature distribution.	Allows for risk from relationships modelled non-linearly to be compared. Gives information on how the population are responding to more ‘extreme’ temperatures. If RR compared are derived from percentiles of the temperature distribution then these can be calculated relative to the whole time period of data (in which case similar to fixing an exact temperature as in a) or allowed to shift (defined relative to each time period analysed). If the percentiles shift according to the time period analysed, this will implicitly include some information about changing susceptibility or ‘adaptation’. If the temperatures used for comparison of RR across the time period are fixed then interpretation of the results is simpler. Results would need to be interpreted with this choice in mind. For example, if the RR at the 98^th^ percentile compared to the average temperature has not changed over time, but the 98^th^ percentile has been calculated according to each time period (i.e. if temperatures have risen, the 98^th^ percentile temperature increases) then this demonstrates some decrease in susceptibility despite no change in RR. Approach a) fixed temperatures or temperatures fixed at a given percentile relative to the entire time period, allows changes in susceptibility to heat/cold to be more easily judged as only one parameter is changed. If approach b) is taken and percentiles used are allowed to vary by time period, then care should be taken in interpreting results. A sensitivity analysis could be undertaken either using approach a) for comparison or by allowing the percentiles used for approach b) both to be fixed across the entire period and allowed to vary.	Approach a) used by Petkova et al. [36] Approach b) used by Astrom et al. [39] who included sensitivity analysis allowing for percentiles at which RR were compared to either vary by time period or be defined relative to the whole time period of data analysis.
Compare deaths attributable to heat or cold over time.	The calculation of attributable deaths takes into account both the threshold above/below which effects are seen and the RR for each temperature above/below the threshold. The calculation of deaths attributable to heat/cold also uses number of days at which the temperature was above/below the threshold for the given period and the baseline mortality for each time period. Therefore the change in outcome could be related to any of these factors which are independent of the temperature-mortality relationship. For example, the RR of heat related mortality may have decreased over time, but the number of days above the threshold increased with as temperatures warm. This may lead to the number of attributable deaths staying constant or increasing over time, despite a decrease in susceptibility compared to the earlier time period. Providing information on the number of days above/below the given threshold or on temperature trends and on trends in baseline mortality may ease interpretation – for example, if the temperature has been increasing but there is a decreasing trend in excess heat-related deaths this gives more weight to evidence of decreasing population susceptibility to heat.	Bobb et al. [37], Carson et al. [44]
Use transfer function (e.g. RR from modelled relationship between temperature and mortality) from later or earlier years with the weather series from whole time period to assess whether there has been a change in attributable deaths.	This approach gives results which are easy to interpret. However, it would need to be made clear whether both the changed RR and potentially changed threshold above/below which effects have been modelled have been used to calculate the burdens. Although using the temperature-mortality relationship from each time period with the same series of weather data seems to give an easily comparable result, clarity should be provided on whether the baseline mortality used for calculations has also been consistent. As for any of the above scenarios, the modelled RR may be influenced by outlying extreme temperatures and therefore taking a number of years as a basis for ‘transfer’ functions may be more reliable.	Christidis et al. [41]

Using excess heat or cold related deaths as an outcome includes many factors: the risk of mortality related to changes in temperature for the given time period, baseline mortality in the population and also the number of days at different temperatures above or below the threshold (where used) within that time period. In studies which used this metric [37, 41, 45, 46], the number of heat-related deaths decreased over time in three studies. However, this could reflect changes in any of the factors mentioned above (e.g. RR, baseline mortality or temperature). It could be expected (though not always reported in these studies) that temperature has been increasing over the last century [1] and therefore the decreasing trend in excess deaths over time in these studies illustrates a decrease in vulnerability despite the increased temperature. One study [41] found that the number of heat related deaths did not decrease over time, but that the regression slope used to calculate these did.

Given that few studies included control for ambient air pollution in the main model it is difficult to know how this would have affected trends. It should be noted that the confounding role of air pollution is currently under debate [50]. In the study by Carson et al. [44], controlling for air pollution did not affect the overall trend over time in cold related mortality, indeed individual RRs for each time period for cold-related mortality were higher after controlling for air pollution. Bobb et al. [37] provided information about models with pollution control as part of a sensitivity analysis. In this paper, when fine particulate matter was included in a linear model, the reduction in heat related mortality between the two time points was no longer significant at the 5 % level (though the reduction remained significant in the non-linear model when air pollution was included).

Influenza is often thought to be a confounding factor when estimating the effects of cold (although whether it is considered a confounder in this relationship will depend on how much influenza survival and transmission rates are affected directly by ambient air temperature (i.e. placing it on the causal pathway between lower temperatures and mortality) as opposed to seasonal and behavioural factors such as school opening times (which occur independently of day to day variation in temperatures)). Three of the five papers reporting cold effects attempted to control for influenza, for example with the inclusion of an indicator for influenza within the models [39] or where flu data was not available by excluding years of known influenza epidemics [42, 44].

#### Variation of effect by subgroup analysis

Where studies examined temperature related mortality by specific subgroups such as cardiovascular or respiratory mortality [37, 38, 43, 44], decreases in these subgroups were seen for the effect of heat and in three of the studies this was significant [36, 37, 43]. Of interest, in the study by Ha et al. [38], there was a (non-significant) decrease in risk of cardiovascular mortality above the temperature threshold in contrast to a (non-significant) increase in all-cause mortality. Carson et al. [44] reported decreases in cardio-vascular and respiratory deaths were less prominent than for all-cause mortality. However, this study analysed a much longer time period than others examining outcome specific mortality and therefore factors such as the epidemiological transition may explain some of the differences. As previously mentioned, this study used weekly data which may also affect the patterns in results seen between different causes of death.

Where results were analysed by age group, the majority of studies found that the largest temporal reductions in mortality were in the older age groups [36, 37, 39]. Barnett [43] and Donaldson et al. [45] only analysed the results in the elderly and over 55s respectively and both found decreases in vulnerability to heat.

#### Variation of effect by location: between and within studies

The variety in approaches used for analysis makes it difficult to compare the variation between studies of effects seen across geographical areas. However, results presented so far have been for area or national level aggregated estimates. Four papers [37, 43, 45, 46] included multiple cities or areas within the same paper (i.e. same methods used). For those which analysed multiple cities within the US [37, 43, 46] some heterogeneity in results was seen. Bobb et al. [37] found that 74/105 cities displayed a significant decrease in excess heat related mortality between 1987 and 2005 and that cities with cooler climates had a larger decline in heat related mortality risk, though these cities also had the highest heat related mortality at the start of the time period. The cities with the largest increase in prevalence of air conditioning over the time period also had the largest declines in mortality, though this was not a statistically significant association. For one city in Southern California, susceptibility increased over time (not statistically significant at the 5 % confidence level). Davies et al. [46] examined 28 US cities over an earlier time period. They found heat related mortality rates had declined in 42 % of the cities but that two cities on the West coast (Seattle and Washington) had an increased number of excess deaths in the later time periods. They also reported that 12 cities (in the South) no longer displayed evidence of a threshold temperature above which heat mortality occurred (see Table 1 for details). Barnett [43] found the largest declines in heat related mortality risk in the US were in the North West, North East, Industrial West and Southern California. The reason for the difference in regional declines seen between these two papers cannot be conclusively determined, though some may be attributable to the difference in levels of aggregation of data (for example, Barnett uses regions, whereas Davies et al.. examine metropolitan areas), the different time periods analysed between studies and potentially the difference in methods used.

Donaldson et al. [45] analysed three different geographical areas (Southern Finland, Northern Carolina and Southern England) and found that the decrease in heat related mortality was smallest in South East England.

#### Susceptibility to extreme temperature events

Six papers were identified that examined differences in all-cause mortality between two different heatwaves or between heatwaves occurring over a number of years in the same location [51–56] (see Table 2 for details). All studies were from high or middle-high income countries. Most of these papers use an episode analysis approach to compare the expected and actual deaths during heatwaves. The approaches taken to selecting an appropriate baseline (for the expected deaths) varied between studies (Table 2) from using a moving 15–30 day average [52] to using more complex models over longer time periods (e.g. [51, 53, 55]). One study compared the absolute number of deaths occurring in two heatwave periods [54]. In comparing different heatwaves, some papers (e.g. [56]) made allowances for the different characteristics of various heatwaves by using model parameters from previous years with weather data from a heatwave in later years and vice versa. Other papers did not make such allowances, but two reported a decrease in heatwave related mortality despite a general increase in the maximum temperature encountered in later heatwaves.

Four papers reported decreased heatwave related mortality in later years [51–54], of which two reported a measure of statistical significance for this. Using a test for linear trend, Kysely et al. [51] found a significant decrease in the effects of heatwaves over the years. Fouillet et al. [53] found the number of deaths to be significantly fewer than those expected when derived from a predictive model based on previous years data.

One study reported no pattern in effects of heatwaves over time [55] and one found a non-significant increase in expected heatwave related deaths in a later year, despite there being an increase in air conditioning over this time and having made allowances for differences in heatwave characteristics [56]. This study used data from Chicago and it was hypothesised that this could be due to the increase in number of persons in the eldest age category between the two events and the number of older persons living below the poverty line (in the US, socio-economic status has been associated with heat related outcomes [57, 58], possibly because it relates to access to working air conditioning which is predictive of reduced heat related mortality [59–61]).

Where a decrease in mortality was seen, potential explanations included the introduction of heat health warning systems (HHWS), increased prevalence of air conditioning, improved urban design and living standards (Table 2). No study attempted to quantify these relationships.

No studies were located that specifically examined the effects of cold snaps over time.

## Discussion

Of the eleven papers that examined variations in the RR of, or heat related mortality over time, all except one [38] found some evidence of decreasing susceptibility. In five of these, this decrease was significant at the 5 % confidence level (either analysed as trend over time or the difference between two discrete time points). Susceptiblity to heat appeared to stabilise over the last part of the century in those studies which covered that time period and in studies analysing more than one location, the magnitude of the decrease varied according to region or city. Where examined, studies found a decrease in cardio-vascular and respiratory heat related mortality.

Comparison of the magnitude of the changes in RR or temperature related mortality between studies is difficult, due to the variety of outcome measures and approaches used to model the temperature-mortality relationships. For example, where thresholds have been used, some studies have fixed temperature thresholds across the whole time period [44] and others have allowed them to vary within time periods analysed [42, 46]. This is important due to the inherent link between the temperature at which the threshold is set and the slope of the exposure-response regression line. There are further inherent limitations of approaches used by individual studies. For example, results of studies which use heat related mortality as an outcome (rather than the RR of death at different temperatures) are also affected by changes in baseline mortality and temperature over time. This can make it difficult to ascertain how much susceptibility to temperature itself is changing over time. Table 3 discusses in more detail the approaches used by individual studies included in this review to assess changes in vulnerability. Whilst we have not gone so far as to recommend one specific approach be used, we do highlight specific aspects of each study design that have implications for the interpretation and comparability of results obtained from these studies (see Table 3). Residual confounding is likely in many of the studies – although the importance of air pollution as a confounder is currently under debate [50], studies examining year round risk also had incomplete control for influenza and other seasonal trends or trends in mortality over time. The results of studies examining temperature related health risks are also aggregated to at least city level, which may lead to a masking of differences in vulnerability of certain population subgroups. It would be important to ascertain, for example, whether different sections of society (e.g. age groups, rural vs. urban populations or groups of different socio-economic status) display differences in their changes in risk of heat related mortality over time. For example, the urban heat island is likely to alter heat related risk and with increasing urbanisation, understanding how urban populations can and have adapted to heat will be important to inform future planning of cities.

**Fig. 5:**
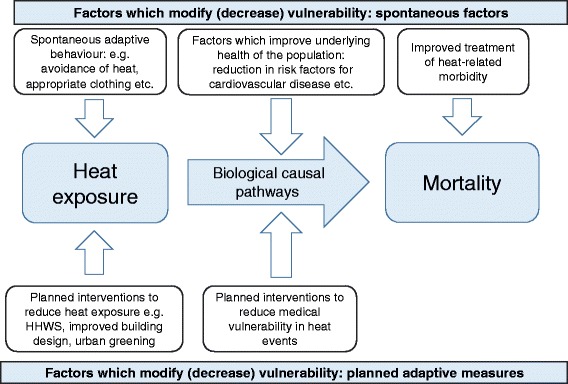
Factors accounting for changes in vulnerability to heat over time

There are also limitations of the body of literature reviewed as a whole. For example, there are no studies specifically from low income settings, where planned adaptive measures may be different to or less prevalent than those used in high income settings. Changes in temperature related mortality over time could be different in these contexts. Secondly, the number of studies is small and, due to differences in outcome measures and approaches used, is difficult to draw conclusions from. Also, many studies [38, 39, 42–45] have not analysed factors contributing to changes in risk over time. Although studies have controlled for general long-term trends in mortality (which should, for example, pick up long term trends in all-cause mortality), whether cause-specific (e.g. cardiovascular) mortality has changed specifically due to adaptation to heat or due to reduced cardio-vascular risk factors in general cannot be determined from the models. Only two papers made an attempt to quantitatively attribute changes in vulnerability to specific adaptive measures [37, 46]. Each found non-significant associations between air conditioning prevalence (see Additional file 1: Table S1b) changes over time and heat related mortality within cities [37] and overall [46]. Other studies included qualitative explanations for the reduction in heat related mortality over time, for example improved urban planning and building design [36, 39, 44, 46], increased living standards and a reduction in risk factors for conditions such as cardiovascular or respiratory morbidity [36]. These possible modifiers of the heat-mortality relationship have been summarised in Fig. 5. Identifying factors which have contributed to such changes could be used to inform environmental and health policy and future urban planning.

The possible slowing of the decline in heat related mortality over the latter part of the last century is interesting. This may, in part, be related to the epidemiological transition (for example, in the later part of the last century, declining susceptibility due to fewer heat related deaths from infectious causes would have occurred but heat-related cardiovascular mortality, for example, may be harder to prevent) but it also potentially demonstrates a limit to ‘adaptation’. For example, there may be limits to both physiological adaptation and adaptive changes in infrastructure. Further studies which examine trends over time and in particular in more recent years are necessary to better understand this. Better integeration of physiological and epidemiological research would enable improved understanding of the importance that physiological adaptation can play within populations.

Overall, studies which have examined the effects of specific heatwave events on mortality over time, have found a reduction of heat-related mortality in later years [51–54]. These studies are not as robust in design as time-series or case-crossover approaches, and particular effects of a given heatwave may vary due to factors not captured in all definitions (e.g. intensity, temperature related to previous days etc.), that is to say that no two heatwaves are the same and have different characteristics which can modify the temperature-mortality relationship [62].

Despite a decreasing vulnerability to heat over time, there is little consistent evidence for decreasing cold related mortality, especially over the latter part of the last century. This may be unexpected, given advancements in housing design and in medical care. However, this should be considered in the context of the small number of studies that examined cold, and fewer that included information on the statistical significance. The lack of reduction in vulnerability to cold remains important as there is some evidence that maximum temperatures are rising faster than minimum temperatures [63]. Conversally, it might be expected by some, that as the climate has warmed over the last century, populations would become less vulnerable to heat and potentially more vulnerable to cold. However, there is no evidence of increased vulnerability to cold, either in terms of cold related mortality or relative risk. This does suggest that, at least at an ecological level, there is no current evidence that ‘maladaptation’ has led to an increased vulnerability to cold over time.

Reasons for the differences over time between heat and cold related mortality have not been quantitatively explained in any papers. Therefore, different explanations should be examined: if improvements in the standard of living and reduction in risk factors for co-morbidities/improved medical care have contributed to some of the temporal decline in heat related mortality, it is reasonable to expect similar reductions in cold related mortality if similar pathways of causation exist. Some of the difference in trend may be due to different causal pathways for heat and cold exposure, for example, cold related mortality is known to occur over longer lag periods and mortality displacement (harvesting) is thought to be less important. It is also hpossible that physiological acclimatisation contributes more substantially to decreasing heat related mortality than to cold related mortality. For example, in their paper, Kysely et al. [51] specifically look at late summer versus early summer mortality from heat waves and find that this decreases over time. Physiological acclimatisation and changes in this over time have not been specifically evaluated in this review and would be an interesting area of further research. As the climate has warmed, the use of air conditioning and heat warning systems/health messaging are also offered as hypotheses for decreased heat related mortality, where these interventions are present. There have also been substantial changes in building design over time. However, whilst some of these might reduce vulnerability to heat specifically, others, such as the increased proportion of people living in flats might be expected to have the opposite effect [64]. Understanding differences in trends between heat and cold related vulnerability represents an important gap in knowledge.

### Evidence from other studies and cities

Studies of differing vulnerability to temperature across geographical regions [21, 24, 26, 65] are often cited as potential evidence for adaptation. A review of these studies [27] used meta-regression to establish city-level characteristics associated with the heat-mortality relationship, demonstrating thresholds were generally higher in communities living closer to the equator. It also found that decreasing GDP, increasing age and population density were associated with increased relative risks of mortality from heat. This evidence is generally consistent with the findings of this review: many of the studies in this review hypothesised that improved standards of living and healthcare would reduce risk factors for disease and also heat exposure, therefore reducing susceptibility to heat over time. It is possible, however, that some cities have become more densely populated which may have increased vulnerability to heat, for example due to higher proportions of the population living in flats and risks of building overheating. However, while comparing results across cities or regions may implicitly include adaptation to temperature over time, it cannot give an estimate of how quickly or by how much community vulnerability can change.

This review provides suggestive evidence of decreasing susceptibility to heat over time. Due to the information included in the studies it cannot, however, determine how much specific adaptive measures (such as the use of cooling systems or HHWS) have contributed to changes compared to general improvements in healthcare and wellbeing in the population. The importance of air conditioning has, however, been demonstrated in other studies [57–61]. Studies, such as one undertaken in migrants which showed reduced vulnerability to heat in those who were born in Southern compared to Northern Italy [66] lend some evidence that physiological and behavioural adaptations to heat could be important and last over population lifespans. Examining trends in cities over time either within the same country or across countries with similar life expectancies and level of development could help further understand the role of adaptation. For example, vulnerability to heat across the US over time was shown to differ by city in the three multi-city studies presented here [37, 43, 46]. Whilst there are likely to be differences in patterns of risk factors and mortality across the US, the overall trend in these factors over time might be broadly expected to be the same. Differences in heat related vulnerability, compared to other specific trends over time by city could support the hypothesis that adaptation to heat specifically has occurred in these areas. Further studies would be required to substantiate this and differentiate different levels of underlying vulnerability across regions.

### Implications for future climate change assessments and policy

A systematic review of future temperature related mortality projections synthesised evidence from 14 studies [67]. Of these, it was found that only half included assumptions about adaptation or changes in vulnerability in future estimates. Methods used to account for adaptation varied from the use of analogue cities [68, 69] analogue summers [70] and assuming adaptation to heat for a pre-determined number of degrees Celsius [71, 72]. The merits and limitations of each of these approaches have been discussed elsewhere [73–75]. Whilst the comparison of vulnerability to temperatures across regions can be used to inform the ‘analogue cities’ approach and differences in early versus late summers can be used to inform how much short term acclimatisation can achieve, the use of past declines in vulnerability has not been used, to our knowledge, to inform any future risk assessments. It could be argued that past trends cannot be used to inform future estimates of adaptation – the climate is projected to warm faster over the next century than in the past [1] and it is uncertain whether future populations will be able to adapt at the same rate (for example, some ‘markets’ for air-conditioning in the US were already thought to be saturated [46]). It is also unknown whether general health gains which lead to reduced vulnerability have been achieved. Nonetheless it is important to recognise that baseline vulnerability to heat in particular has changed across a number of settings. Baseline periods used in a number of studies projecting future temperature related risk studies published over the last decade span a period from the 1960s/70s to the 1990s/2000s [71, 76–78], though some studies - especially those published most recently, have used a more recent baseline period which is likely to improve future estimates [28, 79–81]. Given the trends in mortality observed, estimates of future risk could be improved to better reflect contemporary temperature related health risk. Where this has not been done, projections of future heat related mortality may have been over-estimated.

## Conclusions

There is evidence that the risk of heat related mortality has changed over the last century and more recently. Further studies would be required to improve knowledge in this area, for example to understand the rate of changes in susceptibility more recently and whether changes are occurring at equal rates across sectors of society. Attribution of decreases in mortality to planned adaptive measures may help to inform future actions or policy, as would studies that specifically examine the effectiveness of certain adaptive actions. There are potential policy implications in the lack of decreasing vulnerability to cold. Adaptive efforts should not focus on heat alone, despite warming temperatures. Recent climate change risk assessments (e.g. [28]) show that the risk from cold is expected to account for most of the temperature related risk until late in the century (this is because of the magnitude of the RR and because there remain many more days below cold thresholds until this time). Therefore, any adaptive strategies would ideally reduce the risk from both heat and cold in order to prepare for both short and longer term temperature related risk, and urban and housing design with other co-benefits to health should be emphasised (e.g. [82]). Given the additional risk in urban areas due to the urban heat island effect [83] understanding the risk that future temperatures are likely to pose to health, and how populations can adapt equitably using solutions with co-benefits, is especially important in urbanised societies to plan for healthy and sustainable cities.

Lastly, when considering adaptation in impact assessments of future temperature related risk, sensitivity analyses which include differences in baseline vulnerability could improve understanding of future risk, as would assessments which could include, where possible, effects of certain specific adaptive measures on future heat related risk.

## Additional files

Additional file 1: Table S1a. Features of Studies Examining Changes in Heat and Cold Susceptibility over Time. **Table S1b.** Results of studies examining the change in heat/cold susceptibility over time. (ZIP 50 kb) Additional file 2:
**Peer review reports.** (PDF 62 kb)

## Abbreviations

CRM: Cold related mortality
DF: Degrees of freedom
GDP: Gross domestic product
HRM: Heat related mortality
HW: Heatwave
MMT: Minimum mortality temperature
PAT: Physiological apparent temperature
PM10: Particulate matter ≤10 μm in diameter
RR: Relative risk
