# Supplementary material for: Changes in population susceptibility to heat and cold over time: assessing adaptation to climate change
Source: Environ Health. 2016 Mar 8;15(Suppl 1):33. doi: 10.1186/s12940-016-0102-7 (PMC4895245; doi:10.1186/s12940-016-0102-7)
Supplement: Additional file 1: Table S1a. — Features of Studies Examining Changes in Heat and Cold Susceptibility over Time. Table S1b. Results of studies examining the change in heat/cold susceptibility over time. (ZIP 50 kb) [file 12940_2016_102_MOESM1_ESM.zip › 1476-069X-15-S1-S7-S1a.docx]

Table S1a: Features of Studies Examining Changes in Heat and Cold Susceptibility over Time

| Study | Time period analysed | Population details:  Location  Age | Exposure(s)  assessed and metric | Health outcome(s)  Measure | Modelling approach for baseline estimates | Time varying factors &Lag periods | Sensitivity analyses |
| --- | --- | --- | --- | --- | --- | --- | --- |
| Bobb et al., 2014  [[37](#_ENREF_37)] | 1987-2005 | 105 US cities  All ages  Stratified by age group (<65yrs 65-74 yrs., >75 yrs.) | Heat only (only summer months)  Metric: average daily temperature | Daily all-cause mortality (excluding external causes)  Mortality by cause:  Cardiovascular and Respiratory  Expressed as heat related excess deaths per 1000 deaths | Time series regression (daily series) - 2 stage model:  1)city specific coefficients estimated  2) estimated excess heat related deaths for each year: i.e. each year allowed a different co-efficient on daily temperature (results compared for 1987 and 2005)  City estimates combined to give overall average | Day of week and seasonal variation  Long term trends (natural cubic spline 2df/3months)  Air pollution not in main model (sensitivity analysis)  Lag 0 | Explored the relationship over time constraining it to be linear and without linear constraints  Sensitivity analysis controlling for ozone and fine particular matter presented in supplementary materials.  Note: with control for fine PM and ozone, there remained a significant reduction in heat related deaths in the flexible model, but not in the linear model when controlling for fine PM |
| Petkova et al., 2014  [[36](#_ENREF_36)] | 1900-1948  and  1973-2006 | New York (US)  All ages  Stratified by age group (>15 yrs.,  15-44 yrs.  45-64 yrs.  >65 yrs.) | Heat only (only summer months)  Metric: average daily temperature | Daily all-cause mortality  Expressed as RR of mortality at 29 °C vs 22 °C | Time series regression (daily series)  a) used distributed lag non-linear model to characterise temperature-mortality relationship over the two time periods  b) modelled risk of mortality at 29°C vs 22°C for each decade | Day of week and seasonal variation (quadratic spline - 4 degrees of freedom (d.f.)  Long term trends (natural spline 2 d.f.)  Seasonal variation (natural spline with 4 d.f.)  Air pollution not in model  Lag of 5 days selected. | Tested models with both quadratic and natural cubic splines found quadratic splines gave better model fit. Varied d.f. for quadratic splines.  within-summer  Lags between 3-10 days considered. |
| Astrom et al., 2013  [[39](#_ENREF_39)] | 1901-2009 | Stockholm, Sweden  All ages  Stratified by sex and  age group (0–14yrs, 15–65yrs 65–79yrs 80+yrs ) | Heat and cold ‘extremes’  Temperature extremes defined in main model (model 1) as above/below the 98^th^ percentile for the entire period | Daily mortality  Expressed as RR of mortality at 98th percentiles of temperature compared to mortality at average temperatures | Time series regression (daily series)  Thresholds used as defined  Modelled risk at extremes compared to bassline temperatures for heat and cold | Day of the week and public holidays  long term trend (4 d.f. per year)  binary variable for flu pandemics  binary variable for when data are (in) complete (before/after 1947)  Air pollution not in model | Sensitivity analysis: model 2 presented where extremes were defined as being above/below the decadal threshold rather than the 98^th^ percentile for the whole time frame  Sensitivity analyses of the estimated heat and cold coefficients using 300, 350, 450, and 500 d.f. for the smoothing parameter. |
| Ha et al., 2013  [[38](#_ENREF_38)] | 1993-2009 (exception of 1994 due to extreme heatwave) | Seoul, south Korea  All ages  Stratified by age: analysed data for >65 yrs.  Used direct standardisation to standardise age/sex with the Seoul 2009 population | Heat only  Metric: average daily temperature  Also assessed the effect of hot temperatures in early vs late summer | Daily all-cause mortality (excluding accidental deaths)  Mortality by cause: cardiovascular mortality | Time series regression (daily series)  a)used linear threshold model to analyse quantitative effects – threshold determined by data  b)used natural cubic spline  (NCS) function to assess the temperature–  mortality relationship  Model included  indicator terms for each  year  Used a common threshold temperature value during the study period. | Day-of-week and holiday  Seasonal variation (natural cubic spline function with 3 d.f.)  Long-term trends (15df for 16 yrs.)  Average daily humidity on the current and previous day (0–1 day lag)  Air pollution not in model  Lag 0 | N/A |
| Matzarakis et al., 2011  [[40](#_ENREF_40)] | 1970-2007 | Vienna, Austria  All ages | Heat only  Metric: Physiological equivalent temperatures (PET) | All-cause mortality | Time series analysis (daily series)  Daily excess mortalities compared to baseline mortality /deviations from the average annual mortality: used baseline for average annual deaths calculated for each year to account for changing Life expectancy, etc. | Baseline used: average annual death rates  No explicit control for other time varying short-term factors (e.g. air pollution, day of week etc.) | N/A |
| Christidis et al., 2010  [[41](#_ENREF_41)] | 1976-2005 | England and wales  All ages | Heat and cold  Metric: Central England Temperature | All-cause mortality per million of population | Daily excess heat/cold related mortalities obtained by comparing to the average mortality within a 3 °C ‘comfort zone’  Used optimal detection to also examine contribution of warming climate and anthropogenic emissions under 3 different models : a) ALL - modelled weather data (HadGEM1) with anthropological & other forcing b) anthro - only anthropological forcing in modelled data c) actual data | No explicit control for other time varying short-term factors (e.g. air pollution, day of week etc.) | Sensitivity analyses carried out to check whether year choice affects results |
| Ekamper, 2009  [[42](#_ENREF_42)] | 1855-2006 | Zeeland, Holland  All ages  Stratified by age:  1-4 yes, 20-49 yrs., 50-74 yrs., > 75yrs | Heat and cold  Mean daily temperature | All-cause mortality | Times series analysis (negative binomial distribution) using daily mortality and daily temperature  Thresholds used and allowed to vary for each 25 year time period analysed | Long term trend (using cubic smoothing spline, 7 d.f. per year)  seasonal pattern (using sine and cosine functions)  Excluded years of exceptionally high mortality: e.g. world war 2, 1918 flu epidemic etc.  Results for lags 1-2.3-6, 7-14 and 15-30 days given. | Results from models of each lag period presented. |
| Barnett, 2007  [[43](#_ENREF_43)] | 1987-2000 | 107 US cities  ‘Elderly’ (age range not given) | Increases in temperature in both summer and winter (i.e. examining effects of heat & cold)  Temperature metric not given | Daily Cardiovascular mortality | Case-crossover design  Time stratified | Day of week  Humidity  No influenza/air pollution | N/A |
| Carson et al., 2006  [[44](#_ENREF_44)] | 1900-1996 | London (UK)  All ages | Heat and cold.  Metric: Mean weekly temperature  Health Outcome: | Weekly all-cause mortality  Mortality y cause: cardiovascular and respiratory mortality | Time series regression  Linear hockey stick model using a threshold of 15 °C | Secular trends and seasonality: 7df per year for natural cubic splines  excluded years of war and influenza pandemic  For later period also included air pollution (PM10) | Controlling for air pollution and influenza  For 1992–1996, % increase in mortality per °C below cold threshold was 1.27 (0.86, 1.68) without adjustment for air pollution or influenza, 1.27 (95 percent CI: 0.86, 1.69) controlling for weekly meanPM10, and 1.32 (0.90, 1.75) with additional adjustment for influenza A. In 1954–1964, the figures were 1.64 (1.10, 2.19) without adjustment for pollution, 1.85 (95 percent CI: 1.30, 2.40) with adjustment for particulate pollution measured by the Owen's Smoke Filter, and 1.91 (95 percent CI: 1.30, 2.52) with pollution adjustment and omission of years with high influenza counts. |
| Davies et al., 2003  [[46](#_ENREF_46)] | 1964-1998 | 28 major US cities (Metropolitan Standardised Areas)  Age standardised population | Heat only  Metric: Apparent temperature (AT) | Daily all-cause mortality expressed as excess heat related mortality | Time series analysis:  Analysed daily fluctuations in excess mortality with variation in temperature: converted daily mortality data (age standardised) to daily mortality anomalies by subtracting from each days mortality count the median mortality count for any given month  Threshold for analysis was determined as the AT at which mortality rates were significantly higher than baseline rate. Threshold was allowed to vary between decades.  For each city, excess death rates for all cities above threshold were summed by decade then averaged to give the city specific decadal mean | Monthly fluctuations in death rate captured by using monthly mean as baseline  No explicit control for other time varying short-term factors (e.g. air pollution, day of week etc.) | N/A |
| Donaldson et al.,  2003  [[45](#_ENREF_45)] | 1971-1997 | North Carolina (NC)  SE England (SEE)  South Finland (SF)  Age: over 55 years  Models reported as adjusted for age/sex | Heat only  Metric: daily mean temperature | Daily all-cause mortality expressed as heat related deaths | Time series analysis  3 °C minimum mortality threshold band determined  Excess heat related mortality calculated as: mortalities at daily temperatures which exceeded the 3 °C threshold band, minus the daily mortality in that 3 °C band for the given month. Summed to give annual heat related mortality | Monthly fluctuations in death rate captured by using monthly mean as baseline  No explicit control for other time varying short-term factors (e.g. air pollution, day of week etc.) | With and without controlling for age and sex changes between time periods. |
