# Supplementary material for: Changes in population susceptibility to heat and cold over time: assessing adaptation to climate change
Source: Environ Health. 2016 Mar 8;15(Suppl 1):33. doi: 10.1186/s12940-016-0102-7 (PMC4895245; doi:10.1186/s12940-016-0102-7)
Supplement: Additional file 1: Table S1a. — Features of Studies Examining Changes in Heat and Cold Susceptibility over Time. Table S1b. Results of studies examining the change in heat/cold susceptibility over time. (ZIP 50 kb) [file 12940_2016_102_MOESM1_ESM.zip › 1476-069X-15-S1-S7-S1b.docx]

Table S1b: Results of studies examining the change in heat/cold susceptibility over time

| Study | Methods used to assess change in vulnerability over time | Temperature changes over time (°C) | Health outcomes  (all CI/PIs and significance are reported for 95% level unless stated otherwise) | Explanatory factors where offered for changes in vulnerability |
| --- | --- | --- | --- | --- |
| Bobb et al., 2014  [[37](#_ENREF_37)] | Compared yearly excess heat related mortality over time period:  Each year allowed a separate coefficient for daily temperature but constrained over time to be linear (sensitivity analysis included where no linear constraint) | Not reported | Heat related deaths per 1000 deaths:  Combined results from all cities  1987 51 (95% PI:42,61) compared to 19 (95% PI: 12,27) in 2005  Overall reduction (all cities): 32 (18,45): significant at 95% level  74 of 105 cities displayed reduction in heat related mortality: one area Southern California the risk increased but not significant.  In 18/105 cities temporal decline in mortality was statistically significant  Nationally: excess heat related mortality declined for all age groups (<65,65-75, >_75) but for <65s non-significant  Decline significant for excess heat related respiratory & cardiovascular mortality.  Cities with cooler climates having larger temporal declines though these cities also had larger risks at the beginning of study period.  Temporal trend slowed after year 2000 | The % of homes with AC increased over all 79 cities – by around 1% per year  Cities with larger increases in AC had larger decreases in mortality but association not significant  Using excess deaths as an outcome means daily temperature changes/fluctuations in a given year contribute to magnitude of results so the changes in risk alone are difficult to assess. However, given that temperatures are unlikely to have decreased, this would be expected to increase excess deaths rather than lead to a declining trend  The decrease in cardiovascular/respiratory heat related deaths may in part reflect decreasing risk factors for cardio-respiratory diseases in the population and better care (that are not picked up in the overall trend term included in the model) |
| Petkova et al., 2014  [[36](#_ENREF_36)] | Compared RR at 29°C vs 22°C over time using decadal averages of the RR  Random effects meta-regression, including a linear term for decade | Mean annual temperature(°C)  1900s 11.9  1910s 11.7  1920s 11.8  1930s 12.5  1940s 12.3  1970s 12.6  1980s 12.9  1990s 13.2  2000s 13 | Results of random effects meta-regression showed a decrease in RR at 29°C vs 22°C ( all ages) of 4.6% (2.4,6.7) per decade  Reported more pronounced heat effects on mortality in first part of the century  > 65 yrs.: highest initial risk and experienced most decline over time  Harvesting effect: more prevalent in early part of population | Found a change in lag structure over time - harvesting effect more prevalent in the early part of the century - paper concludes because most vulnerable no form of protection in the earlier part of the century.  Factors hypothesised for decrease in RR:  Improvement in housing conditions, especially through the first part of the century, innovations such as refrigeration, and air conditioning: in 1970, 39% of surveyed households in New York had air conditioning and by 2003 this was 84% households |
| Astrom et al., 2013  [[39](#_ENREF_39)] | Examined trend in RR of mortality at 98th percentiles of temperature compared to mortality at average temperatures. Sensitivity analysis – model 1 vs model 2 (model 1 – temperature extremes defined relative to whole baseline period, model 2 extremes defined relative to decade) | Mean summer temp (°C)  1900s 15.1  1940s 16.6  1980s 16.2  2000s 17.5  Mean winter temp(°C)  1900s —2.2  1940s 3.5  1980s 0.3  2000s 1.7 | Trends similar for men & women  Significant declining trend in temperature related mortality risk for 0-14s for hot and cold  Significant declining trend in risk for elderly and combined age categories for heat but non-significant for cold extremes  In last decades, an upward trend in the heat risk for the 15-64 age group was observed  Models 1 & 2: model 1: generally reports higher estimates of risk than model 2 in the earlier parts of the century. Model 2 reports higher risks in the later part of the century. Potentially due to higher temperatures over time. | Death records around 46% incomplete until 1946 but authors expect variation in day to day deaths the same  Life expectancy in Stockholm over the study period has increased from 56 years to 81 years  No formal/quantitative attribution of declines in heat related mortality to given factors. However, potential explanations include improvements in health sector, urban design and population adaptation  Regarding model 1 and model 2: Model 2 implicitly includes some aspect of ‘adaptation’ to increasing temperatures and accounts for increased temperatures over time |
| Ha et al., 2013  [[38](#_ENREF_38)] | Divided the time series into two  periods (1993 and  1995–2000, and 2001–2009) and included an interaction term for each study period    Used a common threshold temperature value during the study period. | Average daily mean temperatures over 17 year study period: 24.4, 23.1, and 25.7 °C for summer,  early summer, and late summer  No increasing or decreasing trends in  daily mean temperature reported. | Regarding ‘high’ temperatures (above threshold) the % increase in all-cause mortality per 1 °C increase in temperature above threshold:  All-cause mortality  1990s 4.73 % (all ages) 2000s 6.05% (all ages) and 6.78 % (over 65s)  1990ss 6.78 % (over 65s ) 200s 7.89 % (over 65s)  Change not significant at the 95% confidence level  CVD mortality  1990s 8.69 % (all ages) and 2000s (all ages) 5.27 %  1990s 10.47% (over 65s) and 7.29 %(over 65s)  Change not significant at the 95% confidence level  For all summers combined: associations with  higher temperatures are stronger for the > 65 years  and for CVD related mortality than for all-cause-related mortality (all ages). | Proportion of houses with air-conditioning in Seoul increased from 15 % to 71 % between 1994 and 2009  Total health expenditures were also associated related to the observed decline in temperature-related mortality  Declines in temperature-related mortality were particularly noteworthy for late summer: possibility this represents intra-seasonal acclimation improving over time. |
| Matzarakis et al., 2011  [[40](#_ENREF_40)] | linear regressions fitted to the mortality rates per 10 000 to assess % change per decade from 1970 to 2007 in relative mortality for given ranges of PET  t test for the slope of the regression line and non-parametric Mann-Kendall test | Not reported | only the change in relative mortality as a % per decade reported in paper  % change per decade from 1970 to 2007 in relative mortality for each PET range:  <29°C - 0.15%: (CI not given: reported not significant)  29-35°C -0.83% (-0.68,-0.97)  35-41°C -0.96% (-0.77,-1.16)  >=41°C -1.32 % (CI not given: reported not significant - low numbers) | Baseline mortality for the year may take into account some of the expected life expectancy /age structure but likely to have some residual confounding  No age categories, therefore given that age above 60 years decreased from 1970-1990 as a proportion of the population and then increased after that, difficult to remove this effect  No qualitative explanations for why sensitivity might be decreasing given |
| Christidis et al., 2010  [[41](#_ENREF_41)] | 2 comparisons given in paper:  Compared the regression slopes (yearly) obtained from analysis over the time period for relationship between daily mortality per million population and daily temperature: each year from 1976-2005  Change in heat/cold related deaths. Compare actual deaths from latest year with those that would have been obtained using regression slope from earlier years to act as a proxy for ‘adaptation’. | Data showed an average of 0.47°C warming per decade | slope of regression line for heat related mortality risk (SE in brackets)  1976 5.380 (0.574)  1996 2.616 (1.145)  2005 0.866 (0.970)  Slope of regression line for cold related mortality risk (SE in brackets)  1976 -3.437 (0.243)  1996 -2.085 (0.158)  2005 -1.489 (0.112)  Cold related mortality (CRM) decreased by 85 deaths per million per year from 1976-2005. In scenario with adaptation (2005 linear slope used) CRM reduction would have been more moderate: 47 deaths/million/year.  Heat related mortality (HRM) increased by 0.7 deaths per million per year using the 2005 slope but under no adaptation scenario (1976 slope) HRM would have increased by 1.6 deaths per million per year.  no appreciable change in MMT but done as a 3°C band | Sensitivity analyses carried out to check whether year choice affects results  Lack of control for common time varying factors may have led to an over or underestimation of effects |
| Ekamper et al., 2009  [[42](#_ENREF_42)] | Compare regression co-efficient from model between 25 year periods  Compare MMT value in each 25 year time period analysed. | Daily mean Temperature (°C)  1855-79 9.9  1880-1904 10  1905-29 10.1  1930-1954 10.1  1955-1979 9.9  1980-2006 10.7  Number days with max temp > 25°C  1855-79 9  1905-29 7.2  1955-1979 7.7  1980-2006 15.8 | Regression coefficients for heat related mortality  Reported as decreasing over time – no test for significance: pattern apparent at lag 0  Pattern unclear (for a decreasing trend in regression co-efficient) for cold  Decrease in heat related mortality reported to be start to disappear after 1930  Found shift in MMT to higher temperatures in later time periods analysed: MMT slightly below 15 °C for 1855-1897 and around 17°C for 1905-1929 and 1930-1954 | Strongest temperature mortality relationships were for unskilled workers |
| Barnett, 2007  [[43](#_ENREF_43)] | Compare the % increase in of cardiovascular deaths per 10 °F increase in temperature within a given season and across the time period 1987-2000 | Not reported | % increase in risk per 10 °F rise in temperature  summer  1987 4.7% (3.0, 6.5%)  2000 -0.4% (-3.2,2.5)  Winter  1987 -4.2 (-5.1,-3.2)  1987 -4.9 (-6.8,-3.1)  Change in deaths by geographical region: biggest declines in NW, NE, Industrial Midwest and Southern California: also the regions with highest summer mortality in 1987  Winter - little change over time in all regions except in Industrial Midwest where mortality risk with cold got worse, and in Southern California where it improved | Summer time- increase in temp associated with significantly smaller increase in mortality over the time period - hypothesised due to air conditioning or changes in health care.  Winter time - little change in temperature and death relationship - hypothesised that improvements in standard of living were either not useful in decreasing deaths or not sufficient  Effect of not controlling for influenza in winter time deaths in CVD mortality is unclear. There is some evidence that influenza vaccine is associated with a decreased risk of acute cardiovascular events but not conclusive (Warren-Gash et al. 2009) |
| Carson et al., 2006  [[44](#_ENREF_44)] | Compared  a) decadal RR and heat and cold related mortality over the time period and  b)proportion of deaths attributable to heat/cold over the time period  Threshold fixed at 15 °C for whole time period. | Annual mean temperature(°C)  1900-1910 10  1927-1937 10.4  1954-1964 9.6  1986-1996 10.8  1900-1910 10  1927-1937 10.4  1954-1964 9.6  1986-1996 10.8 | RR (CI ) for heat related mortality and % attributable deaths  1900-1910 1.02(-0.16,.21) 0.4% (-0.06,0.86)  1927-1937 1.53(0.152.93) 0.89%(0.09,1.69)  1954-1964 0.29(-1.95,2.59) 0.06% (-0.39,0.5)  1986-1996 -1.34(-1.94,-0.75) -0.9%(-1.31,-0.5)  RR (CI) for cold related mortality and % attributable deaths  1900-1910 2.52 (2, 3.03) 12.5%(10.1,14.9)  1927-1937 2.34 (1.72,2.96) 11.2%(8.4,14.0)  1954-1964 1.64 (1.10,2.19) 8.74%(5.93,11.5)  1986-1996 1.17(0.88, 1.45) 5.42% (4.13,6.69)  Threshold fixed at 15 °C for whole time period. This was best fit with the data for the first 3 decades but for the 19 °C was a better fit | As using weekly data for analysis results for heat may have been attenuated, especially over the last half of the century when heat related deaths may have been less due to GI/bacterial causes. Likely less relevant for cold as less prone to harvesting  Found decreasing vulnerability to heat/cold despite increasing elderly population. Epidemiological transition  For all causes of heat related death and for Cardiovascular/respiratory the general pattern is that the RR is lowers in the last decade: 1986-1996 but highest in the period 1927-1937. However for non-cardio-respiratory deaths there is a more convincing pattern of decline – possibly because at the beginning of the century more heat related deaths were due to GI/diarrheal causes. |
| Davies et al., 2003  [[46](#_ENREF_46)] | Compared excess heat related mortality across decades  Threshold above which heat effects were calculated was allowed to vary between decades. | Summertime trends in Apparent Temperature between 1964-1998:  Significant increase in 9/28 cities (mostly in the Southern United States). In 19 other cities there was a trend of increasing AT but it was not significant. | Excess heat related deaths (+/- SE) in standard population of 1 million  1964-1966 & 1973-1979 41.0 (4.8)  1980-89 17.3(2.7)  1990-1998 10.5 (2.0)  By 1980s, mortality rates declined in 41% of the cities that had elevated mortality a decade earlier  Cities in the Northeast and Great Lakes had remaining excess deaths in 1990s but some decline  Cities in West Coast - Seattle, Washington - actually increased excess deaths in the latest decades compared to the 1960s  12 cities showed no evidence of threshold AT above which heat related mortality begins to appear, in the 1990s. These were mostly in the South and SE. | Likely to be residual confounding from incomplete inclusion of time varying factors in model (mean monthly mortality used as baseline).  Air conditioning prevalence for all regions except one increased during 80s-90s. Excluding the one region without air conditioning statistics- excess heat related mortality decreased by 1.14 deaths/year (per standard million population) for every one % increase in home air conditioning  Some areas in the Us have reached 100% air con saturation  Hypothesised (qualitative) reasoning given by authors for decreasing mortality:  Improved urban planning and architecture, biological acclimatisation air conditioning |
| Donaldson et al.,  2003  [[45](#_ENREF_45)] | Analysed 2 factors:  Change in temperature at which minimum mortality occurs  Change in excess heat related mortality per 10^6 between 1971 and 1997  Regression analysis undertaken with the year as an explanatory variable | Changes in mean summer temperature between 1971-1997 (°C) (in brackets 95% CI for change):    Northern California +1°C (0,2)  South Finland 0°C (-1.5, 1,5)  SE England +2.1°C (0.3,4.0) | Changes in Minimum Mortality Temperature over time:  MMT : 1971 t then change between 1971 and 1996 first without control for age/gender second with control  Northern California: MMT 25.3 °C in 1971, increased by 3.6 °C (significant at 5% level)  South Finland 15.2°C increased by 1.3 °C (not significant)  South East England 18.0°C increased by 2.7 °C (significant at 5% level)  Changes in Excess heat related deaths per 10^6 unadjusted& adjusted for changes in age/sex:  NC in 1971: 228 (140,317) decreased to 16 (-74,104) in 1996 representing a: decrease of 212 (59,365). Adjusted for age/sex decrease of 552 (significant at 95% level)  SF in 1971 382 (257-507) decreased to 99 (-26,225) in 1996 representing a change of 282 (66-500). Adjusted for age/sex change of 414 (significant at 95% level )  SEE In 1971: 111(41,180) decreased to 108 (41,176) in 1996 representing a decrease of 2.1 (-119, 114). When adjusted for age/sex the decrease was 53 (significant at the 95% level) | Likely to be some residual confounding as taking monthly mean deaths as the baseline unlikely to have controlled for all other time varying factors other than temperature) |
