# Supplementary material for: Changes in population susceptibility to heat and cold over time: assessing adaptation to climate change
Source: Environ Health. 2016 Mar 8;15(Suppl 1):33. doi: 10.1186/s12940-016-0102-7 (PMC4895245; doi:10.1186/s12940-016-0102-7)
Supplement: Additional file 2: — Peer review reports. (PDF 62 kb) [file 12940_2016_102_MOESM2_ESM.pdf]

Reviewer 1

The authors reviewed studies which have specifically examined variations in temperature related mortality risks over the 20th and 21st centuries and tried to determine whether population adaptation to heat and/or cold has occurred. They concluded that there is population susceptibility to heat and heatwaves has been decreasing whereas a similar decrease in cold related mortality was not shown. I think their conclusions are generally consistent with widely accepted hypotheses in the environmental epidemiology area. I found that this manuscript is very informative and well organized. I don't have major comments, but a few minor comments.

1. I think adding explanations of RR at all figures(2-4) would be better. For example, RR associated with 1 degree Celsius. Self-explanatory figures would be good.
2. Adding effects of influenza would be good at page 11 just after the statements of ambient air pollution, first paragraph. I think influenza would be a strong confounder for estimating effects of cold temperature on mortality.
3. Two statements on page 4, "In a random-effects meta regression of studies, the relative risk of heat related mortality was found to increase in countries closer to the equator (with higher summertime mean temperatures) and ...." and page 11, "A review of these studies [23]used meta-regression to establish city-level characteristics associated with the heat-mortality relationship, demonstrating thresholds were generally higher in communities living closer to the equator." seem to be contradictory. On pare 4, high-temperature area has higher RR but higher threshold for high-temperature area. Higher threshold generally means smaller vunerability because people suffer for smaller window of temperature range.
4. I think risk of high temperature has been decreasing over years. But the rate of change has also been decreasing. It will eventually saturated to a constant because of the physiological as well as infrastructural limits. Please comment on this idea.

Reviewer 2

EnvironmentalHealth1476-069X-14-S1-S7 manuscript.pdf review

Comments to the editor

I think the authors conveyed important messages out of the reviewed papers. Especially it is very important that no study demonstrated that winter excess mortality will increase along with the global warming. For this reason, I think this paper should be published.

That said, I was a bit frustrated to read this review paper, because they just list up differences among the studies; though I understand that the part of the problem would be diverse methods, observation periods and areas. This may be just my complaint, but if the editor thinks the same way and has ideas to improve this situation, letting them know the ideas would be helpful for the readers.

Comments to the authors

This article reviews the changes in population susceptibility to the effect of cold/heat. The authors' conclusions include very important implication to the future evaluation of net impact of global warming; no study demonstrated that winter excess mortality will increase along with the global warming.

I have some suggestions for improvement below:

1) Putting reference numbers in the first column of each table should greatly improve the readability of this review, because there are only reference numbers in the main text and the readers have to search which reference numbers correspond which studies listed in the tables.

2) Because this is a review paper, external consistency is more important than statistical significance. In this regard, I think the authors put too much weight on statistical significance. For example, in p.11, the

last sentence of the first paragraph, they mentioned only statistical significance. The effect size and its alteration after adding air pollution term would be more of interest for the readers. I would suggest to revise the whole manuscript along this line.

3) Ref 41 (Carson et al.) is the only study in which the unit of observation was week, instead of day, and this fact may have contributed to the difference of this study as shown, for example, in Figure 2. The interpretation of the authors of [41] were introduced, but I believe that inability of obtaining the similar pattern of the heat effect shown by studies with daily data when the weekly data were used should be mentioned.

4) Regarding the two types of the studies, i.e., moving minimum mortality temperature (MMT) and fixed MMT, the authors just described the difference and abandoned to think further. However, given that the assumption of fixed MMT would make the cold risk appear increasing and the heat effect decreasing, there would be room for discussion in comparing these two types of studies. Let me explain using the hypothetical example. The blue 3 V-shapes are the moving MMT type results. If the fixed MMT is assumed, the middle MMT can be used for all the 3 periods. So, the relation of the earliest period would be like the solid red V and that of the latest period would be like the dotted red V. In this example, the moving MMT study find no risk alteration but the fixed MMT study can.

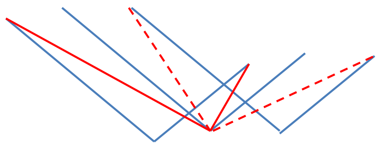

5) p.2 Introduction, first paragraph: "It is anticipated that there will be increasing variability in future temperatures and extreme weather events over most geographical regions [2,3]" The ref 2 and 3 are rather old, and this should be ascertained with newer reference(s) such as ref 1.

6) p.12, Variation of effect by location: between and within studies, the first paragraph 4th line from the bottom

(see Table 2 for details) ... should be Table 1.

7) p.12, Variation of effect by location: between and within studies, the first paragraph

Davies et al [37] showed West cities risk increased over time, whereas Barnett [36] found the decline in the North West. These two studies used the same datasets, I guess, and this contradictory result deserves to be mentioned and discussed.
